# Anti-inflammatory activity of nanocrystalline silver-derived solutions in porcine contact dermatitis

**DOI:** 10.1186/1476-9255-7-13

**Published:** 2010-02-19

**Authors:** Patricia L Nadworny, JianFei Wang, Edward E Tredget, Robert E Burrell

**Affiliations:** 1 Department of Chemical and Materials Engineering, University of Alberta, W7-002 ECERF, Edmonton, Alberta, Canada; 2 Department of Biomedical Engineering, University of Alberta, 1101 Research Transition Facility, Edmonton, Alberta, Canada; 3 Department of Surgery, University of Alberta, Edmonton, Alberta, Canada

## Abstract

**Background:**

Nanocrystalline silver dressings have anti-inflammatory activity, unlike solutions containing Ag^+ ^only, which may be due to dissolution of multiple silver species. These dressings can only be used to treat surfaces. Thus, silver-containing solutions with nanocrystalline silver properties could be valuable for treating hard-to-dress surfaces and inflammatory conditions of the lungs and bowels. This study tested nanocrystalline silver-derived solutions for anti-inflammatory activity.

**Methods:**

Inflammation was induced on porcine backs using dinitrochlorobenzene. Negative and positive controls were treated with distilled water. Experimental groups were treated with solutions generated by dissolving nanocrystalline silver in distilled water adjusted to starting pHs of 4 (using CO_2_), 5.6 (as is), 7, and 9 (using Ca(OH)_2_). Solution samples were analyzed for total silver. Daily imaging, biopsying, erythema and oedema scoring, and treatments were performed for three days. Biopsies were processed for histology, immunohistochemistry (for IL-4, IL-8, IL-10, TNF-α, EGF, KGF, KGF-2, and apoptotic cells), and zymography (MMP-2 and -9). One-way ANOVAs with Tukey-Kramer post tests were used for statistical analyses.

**Results:**

Animals treated with pH 7 and 9 solutions showed clear visual improvements. pH 9 solutions resulted in the most significant reductions in erythema and oedema scores. pH 4 and 7 solutions also reduced oedema scores. Histologically, all treatment groups demonstrated enhanced re-epithelialisation, with decreased inflammation. At 24 h, pMMP-2 expression was significantly lowered with pH 5.6 and 9 treatments, as was aMMP-2 expression with pH 9 treatments. In general, treatment with silver-containing solutions resulted in decreased TNF-α and IL-8 expression, with increased IL-4, EGF, KGF, and KGF-2 expression. At 24 h, apoptotic cells were detected mostly in the dermis with pH 4 and 9 treatments, nowhere with pH 5.6, and in both the epidermis and dermis with pH 7. Solution anti-inflammatory activity did not correlate with total silver content, as pH 4 solutions contained significantly more silver than all others.

**Conclusions:**

Nanocrystalline silver-derived solutions appear to have anti-inflammatory/pro-healing activity, particularly with a starting pH of 9. Solutions generated differently may have varying concentrations of different silver species, only some of which are anti-inflammatory. Nanocrystalline silver-derived solutions show promise for a variety of anti-inflammatory treatment applications.

## Background

Nanocrystalline silver dressings were originally introduced as antimicrobial burn dressings about a decade ago. Studies have since suggested that these dressings have pro-healing and/or anti-inflammatory activity in infected wounds, rashes, and meshed skin grafts[1-5]. Recently, studies have shown that, unlike solutions that contain only Ag^+^, nanocrystalline silver has anti-inflammatory activity independent of its antimicrobial activity in a porcine model of contact dermatitis[6]. Visual and histological signs of inflammation were reduced, apoptosis was induced in inflammatory cells of the dermis, and expression of gelatinases and pro-inflammatory cytokines transforming growth factor (TGF)-β, tumor necrosis factor (TNF)-α, and interleukin (IL)-8 were also reduced[6]. A more recent study has suggested that this effect may be translocatable or systemic, although the effect was weaker with treatment away from the site of injury relative to direct treatments[7]. Another study has shown that, in a murine model of ulcerative colitis, proprietary nanocrystalline silver nanodispersions in polyvinyl alcohol/water delivered intracolonically or orally (at 10 times the dose) suppressed the expression of matrix metalloproteinase (MMP)-9, TNF-α, IL-1β, and IL-12[8]. This suggests that nanocrystalline silver has anti-inflammatory activity which could be used to treat internal epithelial tissues, as well as the skin.

The anti-inflammatory activity of nanocrystalline silver may be due to its small grain size and polycrystallinity, which together result in a high percentage of high energy grain boundaries and defect structures from which unique silver species can dissolve into aqueous solution[9]. One of these unique species released into solution is Ag^0^, which is likely released in a cluster form[9]. Ag^0 ^is the most likely species to have anti-inflammatory activity, as other noble metals have demonstrated similar activity[10-14].

While the anti-inflammatory activity of nanocrystalline silver appears to be potent[6], in its current configuration direct nanocrystalline silver dressing applications are limited to treatment of surfaces, and even in surface applications, tissue contact can be problematic. Since nanocrystalline silver appears to be active via its dissolution products, it is possible that silver-containing solutions could be generated which have some or all of the properties of the nanocrystalline silver dressings. Solutions with these properties would be valuable for anti-inflammatory/pro-healing medical applications including treatment of hard-to-dress surfaces, such as tunnelling wounds, and inflammatory conditions of internal epithelial tissues including the lungs (e.g. acute respiratory distress syndrome) and the gastrointestinal tract (e.g. inflammatory bowel disease). The purpose of this study was to test solutions, derived from nanocrystalline silver under various conditions, for anti-inflammatory activity in a known model of inflammation.

This study shows that nanocrystalline silver-derived solutions have anti-inflammatory and pro-healing properties in the model chosen, as treatment with these solutions resulted in visual and histological improvements. These improvements corresponded to reduced inflammatory cell infiltration (due to apoptosis induction specific to these cells), decreased expression of MMPs and pro-inflammatory cytokines TNF-α and IL-8, and increased expression of anti-inflammatory cytokine IL-4 and epidermal growth factor (EGF), keratinocyte growth factor (KGF, also known as fibroblast growth factor (FGF)-7), and KGF-2 (also known as FGF-10). Activity varied with the conditions under which the silver-containing solutions were generated, but did not correlate with total silver dissolved.

## Methods

### Materials

Silver-containing solutions were generated as follows: Nanocrystalline silver dressings (Acticoat™, Smith and Nephew PLC, Largo, FL) were added at a ratio of 1 in^2^/mL to the following solutions: distilled water (pH 5.6 solution); distilled water adjusted to a pH of 4 by bubbling carbon dioxide through the water (pH 4 solution); distilled water adjusted to a pH of 7 by adding calcium hydroxide drop-wise (pH 7 solution); or distilled water adjusted to a pH of 9 by adding calcium hydroxide drop-wise (pH 9 solution). Containers were sealed and dissolution was allowed to proceed for 24 hours at room temperature under stirring at 100 rpm prior to use.

### Animals

18 young domestic, commercially produced, Large White/Landrace swine (15-20 kg) were used in this study. The animals selected were healthy and without significant wounds or scars on their backs. The animals were kept in individual pens at the Swine Research and Technology Centre (Edmonton, AB) with a 12 hour light/dark cycle, where they were allowed to acclimatize seven days prior to starting experiments. Three animals were used in all experimental groups. The animals received antibiotic-free water and hog ration *ad libitum *during the first three weeks of the experiment. Rations were limited prior to procedures on Days 0 through 3. The study was approved by the University of Alberta Animal Policy & Welfare Division of the Research Ethics Office (formerly Health Sciences Animal Policy and Welfare Committee) and was conducted with humane care of the animals in accordance with guidelines established by the Canadian Council of Animal Care (CCAC).

### Sensitization to DNCB and elicitation of inflammatory reaction

Inflammation was induced using dinitrochlorobenzene (DNCB), similar to procedures described in the literature[1,6,15-17]. On Day -14, the hair on the left side of the backs of 15 pigs was shaved using electric clippers. 10% DNCB (in 4:1 acetone:olive oil) was painted over an area of approximately 15 cm × 25 cm on the shaved portion of the back, which was caudal to the scapula running over the rib cage and five centimetres off the dorsal median line. The total body surface area painted was about 5%, as determined by the equation of Kelley *et al*. [18] The volume of DNCB painted per pig was 3 mL on average. This procedure was repeated on Days -7, -3, and 0. On Day -1, pigs were given transdermal fentanyl patches on shaved skin away from the rash, to avoid discomfort to the pigs during the final application and treatment. The remaining three pigs, which were used as negative controls, were left unexposed to DNCB, but were shaved and received fentanyl patches on Day -1.

### Treatment

Four hours after the final application of DNCB, treatment was commenced with the pigs being placed under general anaesthetic. On Day 0, visual observations were made and 4 mm biopsies were obtained towards the front of the rash (cephalic region), but well within the border of the rash, to ensure that the biopsies were taken from areas which had received good DNCB contact. On subsequent days, biopsies were taken in a line towards the rear of the pig, spaced sufficiently far apart that the new biopsies would not be affected by the previous biopsies, and would still be well within the border of the DNCB-painted area. Calcium alginate dressings were used to reach haemostasis after biopsies were taken. The pigs were then treated. Three positive controls (with rashes) and the three negative controls were treated with distilled water-soaked rayon/polyester gauze. Three pigs were each treated with gauze soaked in pH 4, pH 5.6, pH 7, or pH 9 silver-containing solutions which were generated as described above. New fentanyl patches were applied, if they had come loose. Surgical drape was placed over each dressing to provide moisture control, and elastic adhesive dressing was used to hold the dressings in place. The procedures of Day 0 were repeated on Day 1 and Day 2 (at 24 and 48 h). On Day 3 (72 h), after visual images, scores, and biopsies were taken, the pigs were euthanized.

### Total Silver Analysis

Samples of nanocrystalline silver derived solutions were obtained daily at the time of treatment, and submitted for total silver analysis by atomic absorption spectroscopy (AAS). For AAS, a Varian 220 FS double beam Atomic Absorption Spectrophotometer was used, with the following instrument parameters: an Ag hollow cathode lamp with a wavelength of 328.1 nm, and a lean air-acetylene flame. A calibration plot was generated using silver standards of 0.5, 1.0, 3.0, and 5.0 ppm, prepared from a silver standard stock solution of 1000 ppm. If the solutions contained more than 5 ppm silver, they were diluted as necessary with distilled water until they were in the linear range for silver analysis (0.1 ppm to 5 ppm).

### Visual observations

Pictures were taken of the rash, with wound rulers included, on each treatment day. Erythema and oedema were graded on a scale of 0-4 on Days 0 through 3 (0, 24, 48, and 72 h), using the following scale: 0 - no erythema or oedema; 1 - barely visible pink, or mildly raised tissue covering parts of the rash; 2 - moderate redness, or moderately raised firm tissue covering parts of the rash; 3 - severe bright red erythema, or obvious swelling and hardness of tissues over most of the rash; 4 - dark red/purple erythema, or hard raised tissue over the entire rash.

### Histopathology

All samples to be paraffinised were placed in 4% neutral buffered paraformaldehyde. The samples were then dehydrated in alcohol and xylene; oriented and embedded in paraffin; and sectioned (5 μm). For histopathological analysis, sections were stained with haematoxylin and eosin following standard procedures[19]. Images were taken of the epidermal-dermal junction (or the surface of the tissue if there was no clear junction due to tissue damage caused by the rashes) for each animal at each time point at 100× magnification using an optical microscope with an attached digital camera.

### Gelatinase zymography

Gelatinase activity was measured similar to the methods used previously, with some minor modifications[6]. To extract protein, half of a snap-frozen biopsy from each animal was homogenized using a Mikro-Dismembrator (B. Braun Biotech International, Allentown, PA, USA) for 30 seconds at 2600 rpm. 1 mL of lysis buffer (1% Triton-X 100, 20% glycerol in phosphate buffered saline (PBS)) was added to the samples for protein extraction. Homogenates were centrifuged at 13 000 rpm for 30 minutes at 4°C to remove debris. Total protein concentrations were measured with a BCA protein assay reagent kit (Pierce Biotechnology, Inc., Rockford, IL, US). Protease activity was then measured using gelatine zymographs[20], using the same protein concentration for each sample. To run the zymogram, 12% polyacrylamide gels (1.5 mm thick) were cast containing 0.15% gelatine. Samples were applied to the gels under non-reducing conditions without heating. After running the gels, they were rinsed in 2% Triton X-100 on a gyratory shaker (0.5 h, room temperature), incubated in developing buffer (50 mM Tris pH 8.0, 0.1 mM CaCl_2_) overnight at 37°C, and stained with Coomassie blue. Excess stain was removed using a destaining solution (50 mL acetic acid, 200 mL methanol, 250 mL ddH_2_O). Gelatinase activity appears as a clear band (indicative of cleavage of the gelatine substrate) on a blue background. For quantitative analysis, photographs of the gels were loaded into AlphaImager software (AlphaEase, FC Software Version 4.1.0, Alpha Innotech Corporation, San Leandro, CA, USA © 1993-2004). The integrated density value (IDV) of each band was measured, holding the band area constant. Each IDV was then divided by the IDV of a portion of the gel background of the same area, to correct for differences in gel densities between the four gels required to run all the samples.

### Apoptosis detection

Detection of the presence of apoptotic cells in tissue samples after 24 hours of treatment was determined using the In Situ Cell Death Detection Kit (Roche Applied Sciences, Basel, Switzerland), as described previously[6], with modifications. Briefly, paraffinised tissue samples were dewaxed, rehydrated, and treated with proteinase K (25 μg/mL) for half an hour at 37°C. Tissues were then incubated at 4°C overnight with fluorescein isothiocyanate (FITC)-labelled deoxyribonucleotide triphosphate (dNTP) and terminal deoxynucleotidyl transferase (TdT). The tissue samples were mounted using a polyvinyl alcohol based mounting medium containing 1:1000 4',6-diamidino-2-phenylindole (DAPI, provided by the Department of Oncology Cell Imaging Facility, University of Alberta) for nuclear counterstaining. Sections were imaged using a Zeiss LSM510 multi-channel laser scanning confocal microscope (Carl Zeiss MicroImaging GmbH, Oberkochen, Germany) at the Cell Imaging Facility. Images were taken using the following settings: objective: 40× 1.3; laser for DAPI: 364 nm, 1% power, 444 μm pinhole; and laser for FITC: 488 nm, 4% power, 91 μm pinhole. Images were taken of the deep dermis and of the epidermal-dermal junction, which was taken to be either where re-epithelialisation was occurring or to be the tissue surface, if no re-epithelialisation was observed in the tissue. Images selected to represent each group were median images in terms of their apoptotic staining. Semi-quantitative analysis was performed using ImageJ software (Rasband, W., v1.37, NIH, Rockville, MD, USA. © 2007). First, the epidermis or dermis was manually selected. An AND function was used to select only apoptotic staining which was colocalized with nuclear staining, in order to eliminate any background staining. The same thresholds were used for all samples, since they were stained and imaged under identical conditions. Total numbers of green (apoptotic staining) and blue (nuclear staining) pixels were counted, and a ratio of green to blue pixels was calculated to obtain a relative measure of apoptotic activity. Images in which apoptotic staining did not coincide with nuclear staining were excluded.

### Immunohistochemistry

Tissue samples after 24 h and 72 h of treatment were analyzed for the presence of TNF-α, IL-4, IL-8, IL-10, EGF, KGF (FGF-7), and KGF-2 (FGF-10), as described previously[7]. Briefly, paraffinised samples were dewaxed and rehydrated. To improve antigen retrieval, samples tested for TNF-α, IL-8, and KGF were incubated in 25 μg/mL proteinase K at 37°C for 20 minutes. All samples were then treated with 3% H_2_O_2 _for 30 minutes to quench endogenous peroxidase activity, and then blocked for one hour with the sera from the species that the secondary antibody was raised in (rabbit for KGF, KGF-2, and IL-4; goat for TNF-α, IL-8, IL-10, and EGF). Sections were then incubated overnight at 4°C with 5 μg/mL of the appropriate antibody: mouse-anti-pTNF-α (MP390, Endogen, Fisher Scientific Inc., Ottawa, Ontario, Canada), mouse-anti-pIL-8 antibody (MP800, Endogen), goat-anti-pIL-4 (AF654, R&D Systems, Minneapolis, MN, USA), mouse-anti-hEGF (MAB236, R&D Systems), mouse-anti-pIL-10 (MAB6932, R&D Systems), goat-anti-hFGF-7 (KGF, AF-251-NA, R&D Systems), or goat-anti-hFGF-10 (KGF-2, AF345, R&D Systems). For sections incubated with primary antibodies produced in mouse, negative control tissues were incubated with 5 μg/mL mouse IgG during this step. These sections were subsequently incubated with goat-anti-mouse-HRP (horseradish peroxidase - R&D Systems, 1:400 in 2% pig serum) for one hour. For sections incubated with primary antibodies produced in goat, negative control tissues were incubated with PBS during the primary antibody incubation step. These sections were then incubated with rabbit-anti-goat-HRP (R&D Systems, 1:400 in 2% pig serum). All tissues were then stained using 3,3'-diaminobenzidine (DAB) and H_2_O_2 _(25 mg DAB, 50 μL H_2_O_2 _in 50 mL PBS). Samples were then counterstained with haematoxylin (30 seconds), dehydrated, and mounted using Permount™ mounting solution. Images of the samples were taken as described for histology. Samples stained for one cytokine were run in three batches of twelve slides under identical conditions. Each batch contained samples from all treatment groups. Therefore, the intensity of staining can be used as a qualitative indication of the relative quantity of cytokines present in the tissues. Intensity of staining was scored on a scale from 0 to 4 as follows: 0 - no staining anywhere; 1 - very small areas of staining and/or very light staining; 2 - small areas of dark staining and/or larger areas of light staining; 3 - diffuse light staining and/or larger areas of dark staining, 4 - diffuse dark staining.

### Statistics

Tests were performed on all three pigs from each group to confirm result reproducibility. For numerical results, one-way ANOVAs with Tukey-Kramer Multiple Comparisons post tests were performed using GraphPad InStat version 3.06 (GraphPad Software, San Diego, California, USA, http://www.graphpad.com, © 2003) for normally distributed data. For data which was not normally distributed (apoptotic staining data), Kruskal-Wallis Tests (non-parametric ANOVAs) were performed with Dunn's Multiple Comparisons post-test, also using GraphPad InStat. Standard deviations are plotted as error bars for all data points. For some data points, the standard deviation was very small.

## Results

### Visual observations

**Figure 1:**
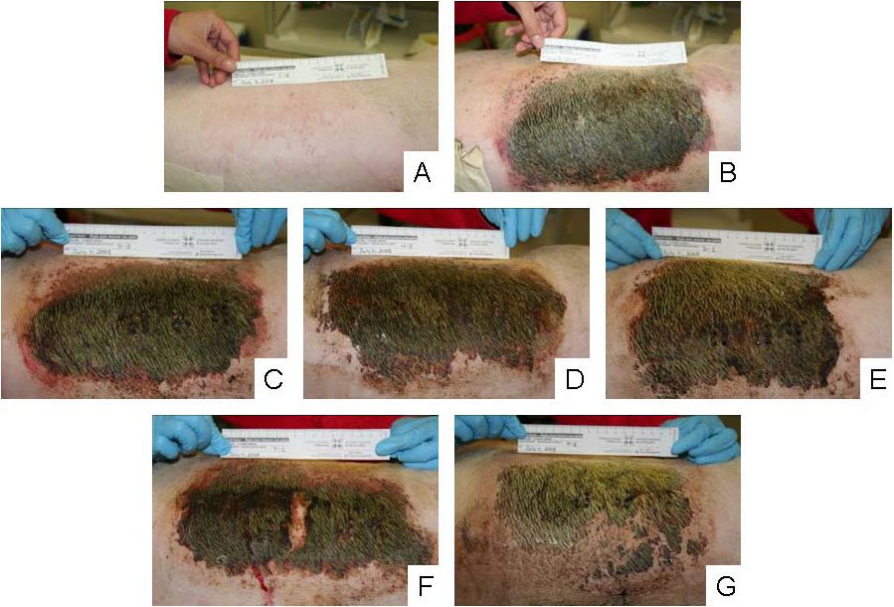
**Digital images of DNCB-induced rashes treated with various nanocrystalline silver-derived solutions**. Representative digital images are shown for (A) negative controls (pigs which received no rash, and were treated with distilled water-soaked dressings); (B) DNCB-induced rashes on Day 0 before treatment was commenced; (C) positive controls (pigs which had DNCB-induced rashes and were treated with distilled water) after 72 hours of treatment; and animals treated for 72 hours with nanocrystalline silver-derived solutions with starting pHs of (D) 4, (E) 5.6, (F) 7, and (G) 9.

Figure 1 shows representative digital images of a negative control (A), a DNCB induced rash just prior to commencing treatment (B), a positive control (rash treated with distilled water) after 72 hours of treatment (C), and animals with rashes treated for 72 hours with pH 4 (D), 5.6 (E), 7 (F), and 9 (G) silver-containing solutions. Animals treated with pH 7 and 9 solutions showed the most improvement during treatment, with decreased redness and swelling around the rash edges, and areas where the scabbing had fallen off, revealing healthy tissue underneath. Animals treated with pH 4 and 5.6 solutions showed some improvement during treatment, with decreased redness around the edges of the rash. However, the scabbing mostly stayed in place for these treatment groups. Positive controls showed little improvement over 72 hours, with a full scab across the rash, and redness and swelling around the scab.

**Figure 2:**
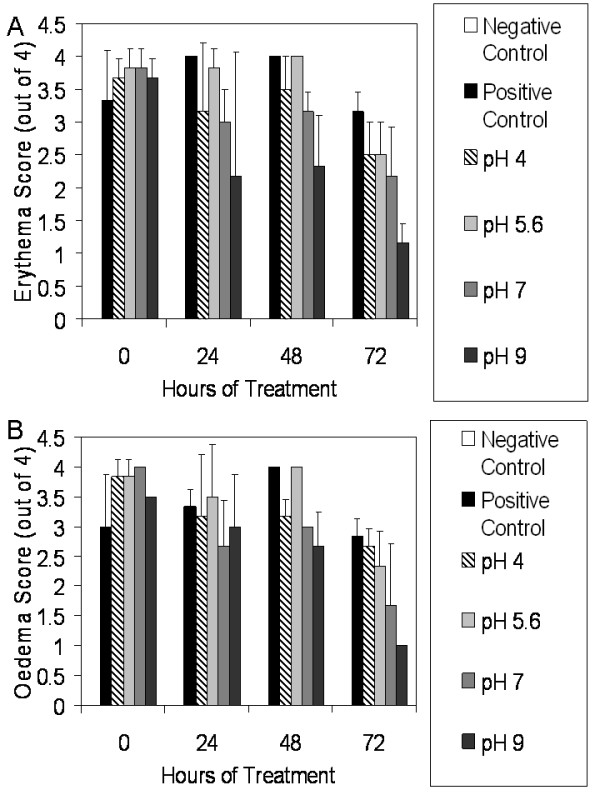
**Erythema and oedema scores for DNCB-induced rashes treated with various nanocrystalline silver-derived solutions**. Daily average erythema and oedema scores are shown in Panels A and B, respectively, for negative controls (pigs without rashes treated with distilled water-soaked dressings), and for pigs with DNCB-induced contact dermatitis treated for three days with distilled water (positive controls) or nanocrystalline silver-derived solutions with starting pHs of 4, 5.6, 7, or 9. The statistical analyses, which were performed using one-way ANOVAs with Tukey-Kramer Multiple Comparisons post tests, are shown in Table 1. Error bars represent standard deviations.

Figure 2A shows the average erythema scores for pigs treated with various nanocrystalline silver-derived solutions relative to positive and negative controls. From 48 hours of treatment on, animals treated with pH 9 solutions had significantly lower erythema scores relative to positive controls and animals treated with pH 4 and pH 5.6 solutions (see Table 1). Figure 2B shows the average oedema scores for pigs treated with various nanocrystalline silver-derived solutions, again relative to positive and negative controls. After 48 hours of treatment, animals treated with pH 4, 7, or 9 solutions had significantly lower oedema scores relative to positive controls or to animals treated with pH 5.6 solutions. After 72 hours of treatment, animals treated with pH 9 solutions had significantly lower oedema scores relative to positive controls and to animals treated with pH 4 solutions (see Table 1).

**Table 1:** Statistical analysis of erythema and oedema scores*.

Assay	Time (h)	ANOVA	Post Test Results
Erythema	0	p < 0.0001	Negative control < Positive Control (p < 0.001)
			Negative control < pH 4 (p < 0.001)
			Negative control < pH 5.6 (p < 0.001)
			Negative control < pH 7 (p < 0.001)
			Negative control < pH 9 (p < 0.001)
Erythema	24	p = 0.0018	Negative control < Positive Control (p < 0.01)
			Negative control < pH 4 (p < 0.05)
			Negative control < pH 5.6 (p < 0.01)
			Negative control < pH 7 (p < 0.05)
Erythema	48	p < 0.0001	Negative control < Positive Control (p < 0.001)
			Negative control < pH 4 (p < 0.001)
			Negative control < pH 5.6 (p < 0.001)
			Negative control < pH 7 (p < 0.001)
			Negative control < pH 9 (p < 0.001)
			pH 9 < Positive Control (p < 0.01)
			pH 9 < pH 4 (p < 0.05)
			pH 9 < pH 5.6 (p < 0.01)
Erythema	72	p < 0.0001	Negative control < Positive Control (p < 0.001)
			Negative control < pH 4 (p < 0.001)
			Negative control < pH 5.6 (p < 0.001)
			Negative control < pH 7 (p < 0.001)
			pH 9 < Positive Control (p < 0.01)
			pH 9 < pH 4 (p < 0.05)
			pH 9 < pH 5.6 (p < 0.05)
Oedema	0	p < 0.0001	Negative control < Positive Control (p < 0.001)
			Negative control < pH 4 (p < 0.001)
			Negative control < pH 5.6 (p < 0.001)
			Negative control < pH 7 (p < 0.001)
			Negative control < pH 9 (p < 0.001)
Oedema	24	p = 0.0007	Negative control < Positive Control (p < 0.01)
			Negative control < pH 4 (p < 0.01)
			Negative control < pH 5.6 (p < 0.001)
			Negative control < pH 7 (p < 0.01)
			Negative control < pH 9 (p < 0.01)
Oedema	48	p < 0.0001	Negative control < Positive Control (p < 0.001)
			Negative control < pH 4 (p < 0.001)
			Negative control < pH 5.6 (p < 0.001)
			Negative control < pH 7 (p < 0.001)
			Negative control < pH 9 (p < 0.001)
			pH 4 < Positive Control (p < 0.05)
			pH 7 < Positive Control (p < 0.01)
			pH 9 < Positive Control (p < 0.001)
			pH 4 < pH 5.6 (p < 0.05)
			pH 7 < pH 5.6 (p < 0.01)
			pH 9 < pH 5.6 (p < 0.001)
Oedema	72	p = 0.0001	Negative control < Positive Control (p < 0.001)
			Negative control < pH 4 (p < 0.001)
			Negative control < pH 5.6 (p < 0.01)
			Negative control < pH 7 (p < 0.05)
			pH 9 < Positive Control (p < 0.01)
			pH 9 < pH 4 (p < 0.05)

*Statistical analyses were performed using one-way ANOVAs with Tukey-Kramer Multiple Comparisons Post Tests. All treatment groups were compared in an ANOVA, and if the ANOVA indicated significant differences were present between groups, each treatment group was compared to every other treatment group in post testing. Only statistically significant post test results are shown. Any treatment group comparisons not listed were not significantly different from one another (p > 0.05).

### Histopathology

**Figure 3:**
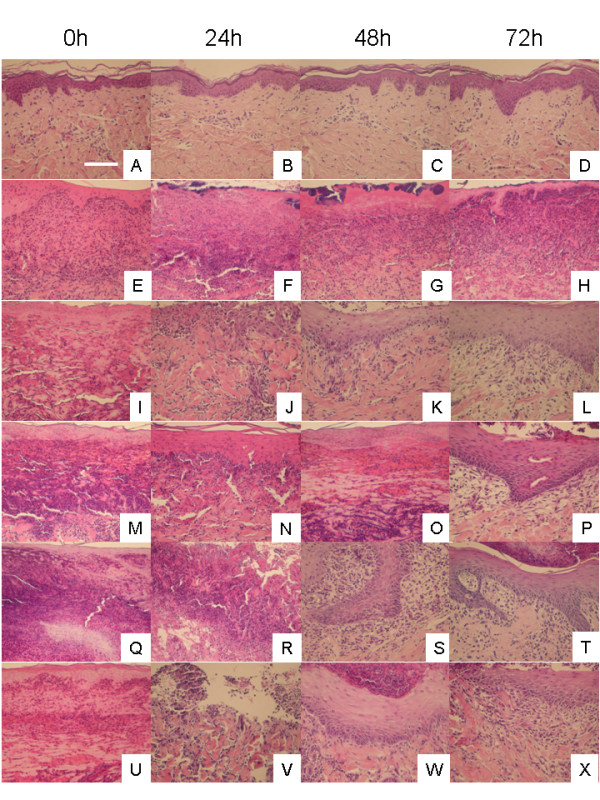
**Representative histological images for DNCB-induced rashes treated with various nanocrystalline silver-derived solutions**. Representative images, including portions of both the epidermis and the dermis, are shown at 0, 24, 48, and 72 h for negative controls (pigs which did not have rashes and were treated with distilled water-soaked gauze) (A-D), positive controls (pigs which had DNCB-induced rashes which were treated with distilled water-soaked gauze) (E-H), and animals with DNCB-induced rashes treated with nanocrystalline silver-derived solutions generated at starting pHs of 4 (I-L), 5.6 (M-P), 7 (Q-T), or 9 (U-X). Cell nuclei were stained purple with haematoxylin, while cytoplasm was stained pink with eosin. The scale bar in A represents 100 μm.

Representative histological images over the course of treatment are shown in Figure 3. Before treatment (column 1), tissues from all DNCB-challenged animals demonstrated leukocyte and red blood cell infiltration, with epidermal disruption due to excessive oedema. Negative controls (A-D) showed normal tissue morphology throughout the study, with a well-defined epidermis, and low cellularity in the dermis. Positive controls (E-H) showed no improvement over the course of treatment, with extensive infiltration of inflammatory cells throughout the experiment. Animals treated with pH 4 solutions (I-L) showed a gradual reduction of inflammatory cells over the course of treatment, with re-epithelialisation beginning to occur by 48 hours of treatment. Animals treated with pH 5.6 solutions (M-P) did not show histological signs of improvement until 72 hours of treatment, at which point decreased inflammatory cells were present, and re-epithelialisation began. Animals treated with pH 7 solutions (Q-T) had begun re-epithelialisation at 48 hours, and showed lower leukocyte infiltration at 72 hours than animals treated with pH 4 solutions. However, the re-epithelialisation that occurred appeared to be thicker with deeper ridges. Animals treated with pH 9 solutions (U-X) all showed signs of re-epithelialisation at 48 hours of treatment, with one animal even showing signs of re-epithelialisation at 24 hours (not shown). At 72 hours, the animals treated with pH 9 solutions showed the best overall tissue morphology, including the most well-defined epidermis and dermis, clearest dermal morphology, and lowest leukocyte infiltration.

### Zymography for gelatinases

**Figure 4:**
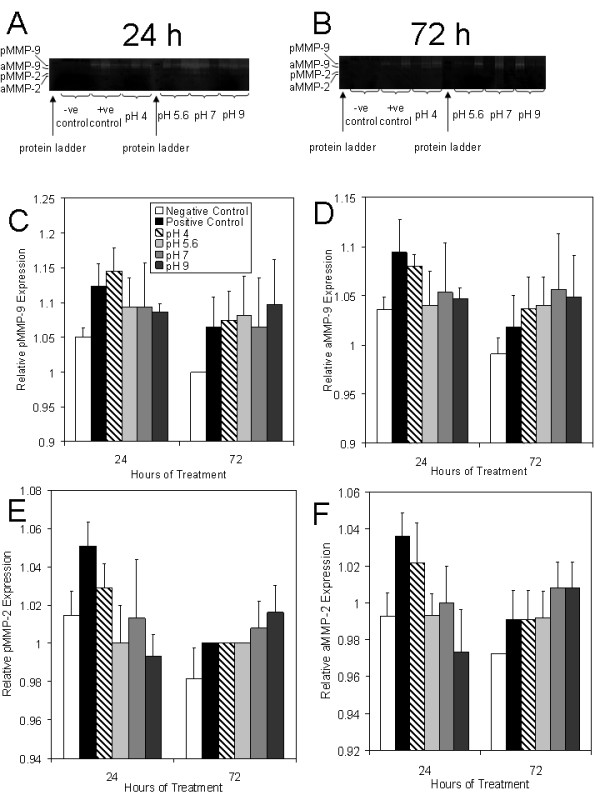
**Gelatinase activity in biopsies from DNCB-induced rashes treated with various nanocrystalline silver-derived solutions**. Zymograms are shown for all three animals of each treatment group at 24 (A) and 72 (B) hours in the following order for each time period: negative controls (no rash, treated with distilled water), positive controls (had DNCB-induced rash, treated with distilled water), and animals with DNCB-induced rashes that were treated with nanocrystalline silver-derived solutions generated at starting pHs of 4, 5.6, 7, and 9. Protein ladders were run as the first sample on each gel. The gels testing biopsies from 24 hours were run simultaneously, as were the gels testing 72 hour biopsies. The integrated density values (IDV) relative to the gel background IDV for pMMP-9, aMMP-9, pMMP-2, and aMMP-2 are shown in Panels C, D, E, and F, respectively. The statistical analyses, which were performed using one-way ANOVAs with Tukey-Kramer Multiple Comparisons Post Tests, are shown in Table 2. Error bars represent standard deviations.

Figure 4 shows zymograms for all pigs in each treatment and control group after 24 (A) and 72 (B) hours of treatment. Throughout treatment, negative controls visually showed very low levels of gelatinases. After 72 hours of treatment, positive controls and pH 4 solution treated animals had two animals out of three showing high gelatinase levels, while animals treated with pH 5.6, 7, and 9 solutions had only one animal out of three showing high gelatinase levels. Figure 4C shows the semi-quantitative analysis of proMMP-9 (pMMP-9) levels, which showed a trend towards significant differences between groups (p = 0.0817), with pMMP-9 levels being lower for pH 5.6, 7, and 9 treatments relative to positive controls and pH 4 treatments (see Table 2 for statistical analysis). All treatment groups showed similar levels at 72 hours. Figure 4D shows the semi-quantitative analysis of active MMP-9 (aMMP-9) levels. Again, there was a trend towards significant differences between groups (p = 0.0944), with lower expression levels at 24 h for silver treated animals relative to positive controls, particularly with treatments at pH 5.6, 7, and 9. Panel 4E shows the semi-quantitative analysis for pMMP-2, which showed significant differences between groups (p = 0.0010), with pH 5.6 and 9 treated animals having significantly lower pMMP-2 levels relative to positive controls after 24 hours of treatment (see Table 2). Panel 4F shows the semi-quantitative analysis for aMMP-2, which also showed significant differences between groups (p = 0.0019), with pH 9 solution treated animals showing significantly lower aMMP-2 levels at 24 hours relative to positive controls and pH 4 solution treated animals (see Table 2).

**Table 2:** Statistical analysis of gelatinase activity*.

MMP	ANOVA	Post Test Results
pMMP-9	p = 0.0817	No significant differences.
aMMP-9	p = 0.0944	No significant differences.
pMMP-2	p = 0.0010	pH 5.6 (24 h) < Positive Control (24 h) (p < 0.05)
		pH 9 (24 h) < Positive Control (24 h) (p < 0.01)
		Negative Control (72 h) < Positive Control (24 h) (p < 0.001)
		Positive Control (72 h) < Positive Control (24 h) (p < 0.05)
		pH 4 (72 h) < Positive Control (24 h) (p < 0.05)
		pH 5.6 (72 h) < Positive Control (24 h) (p < 0.05)
		Negative Control (72 h) < pH 4 (24 h) (p < 0.05)
aMMP-2	p = 0.0019	pH 9 (24 h) < Positive Control (24 h) (p < 0.01)
		pH 9 (24 h) < pH 4 (24 h) (p < 0.05)
		Negative Control (72 h) < Positive Control (24 h) (p < 0.01)
		Negative Control (72 h) < pH 4 (24 h) (p < 0.05)

* Statistical analyses were performed using one-way ANOVAs with Tukey-Kramer Multiple Comparisons Post Tests. All treatment groups were compared in an ANOVA, and if the ANOVA indicated significant differences were present between groups, each treatment group was compared to every other treatment group in post testing. Only statistically significant post test results are shown. Any treatment group comparisons not listed were not significantly different from one another (p > 0.05).

### Apoptosis detection

**Figure 5:**
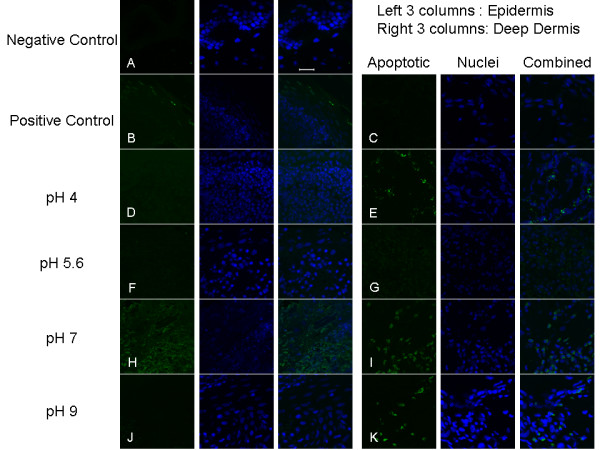
**Apoptosis detection in biopsies of DNCB-induced rashes treated for 24 h with various nanocrystalline silver-derived solutions**. Representative fluorescence images obtained via confocal microscopy for immunohistochemical detection of apoptotic cells in pigs with DNCB-induced rashes after 24 h of various treatments are shown. The first column shows staining by FITC for apoptotic cells (green). The second column shows counterstaining by DAPI for nuclei (blue). The third column shows the combination of apoptotic and nuclear staining. Images in Row A are from the surface (epidermis and upper dermis) of a negative control (no rash) treated with distilled water. Images in Rows B and C are of the skin surface and deep dermis, respectively, of a positive control (rash treated with distilled water). Images in Rows D and E are of the surface and the deep dermis, respectively, of a DNCB-induced porcine rash treated with a nanocrystalline silver-derived solution with a starting pH of 4. Images in Rows F and G are of the surface and the deep dermis, respectively, of a DNCB-induced porcine rash treated with a nanocrystalline silver-derived solution with a starting pH of 5.6. Images in Rows H and I are of the surface and the deep dermis, respectively, of a DNCB-induced porcine rash treated with a nanocrystalline silver-derived solution with a starting pH of 7. Images in Rows J and K are of the surface and the deep dermis, respectively, of a DNCB-induced porcine rash treated with a nanocrystalline silver-derived solution with a starting pH of 9. The scale bar in the far right image in Row A represents 20 μm.

**Figure 6:**
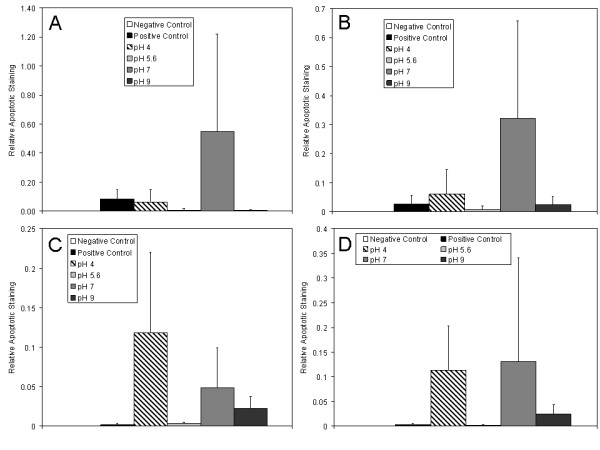
**Semi-quantitative analysis of apoptosis in DNCB-induced rashes treated for 24 h with nanocrystalline silver-derived solutions**. Semi-quantitative analysis of apoptotic staining in biopsies from pigs with DNCB-induced rashes after 24 h of various treatments is shown. The relative apoptotic staining level was calculated by taking a ratio of apoptotic staining (where colocalized with nuclear staining) to total nuclear staining in a given image window. Semi-quantitative analysis of staining in the epidermis, superficial dermis, deep dermis, and all dermal images combined are shown in (A) through (D), respectively. Statistical analysis, which was performed using Kruskal Wallis testing with Dunn's Multiple Comparisons post testing, is provided in Table 3. Error bars represent standard deviations.

Figure 5 shows representative images of staining for apoptotic cells after 24 hours of various treatments. Figure 6 shows a semi-quantitative analysis of apoptotic staining in the epidermis (A), superficial dermis (B), deep dermis (C), and total dermis (D). Table 3 shows statistical analysis of these results. Negative controls (Figure 5A) had very few apoptotic cells. Positive controls showed somewhat higher levels of apoptosis in the epidermis (Figure 5B), but had decreasing levels of apoptosis with tissue depth, with virtually no cells undergoing apoptosis in the deep dermis (Figure 5C, 6C). Animals treated with pH 4 solutions had somewhat lower levels of apoptosis induction in the epidermis relative to positive controls, with similar levels present in the superficial dermis (Figure 5D, 6A-B). However, they demonstrated the highest level of apoptotic cells in the deep dermis (Figure 5E, 6C), with levels significantly higher than negative controls. Animals treated with pH 5.6 solutions did not demonstrate apoptosis induction in either the epidermis (Figure 5F, 6A) or the dermis (Figure 5G, 6B-D). Animals treated with pH 7 solutions showed the highest levels of apoptotic cells in the upper dermis, with significantly higher staining than negative controls (Figure 6B), and in the epidermis as well (Figure 5H), although this did not reach statistical significance due to high interanimal variability (Figure 6A). Apoptotic staining was also present to a lesser extent in the deep dermis (Figure 5I, 6C). Animals treated with pH 9 solutions did not show apoptotic staining in the newly forming epidermis (Figure 5J, 6A), but did have apoptotic cells in the dermis, although to a lesser extent than present in the pH 4 and 7 treated animals (Figure 5K, 6B-C). Combining the superficial and deep dermal semi-quantitative staining results, animals treated with pH 4 solutions had significantly higher apoptotic staining than negative controls, positive controls, and pH 5.6 solution-treated animals, while animals treated with pH 7 solutions had significantly higher apoptotic staining relative to negative controls and pH 5.6 solution-treated animals (Figure 6D).

**Table 3:** Statistical analysis of apoptotic staining*.

Skin Layer	Kruskal-Wallis	Post Test Results
Epidermis	p = 0.0673	No significant differences.
Superficial Dermis	p = 0.0433	Negative Control < pH 7 (p < 0.05)
Deep Dermis	p = 0.0055	Negative Control < pH 4 (p < 0.05)
Whole Dermis	p < 0.0001	Positive Control < pH 4 (p < 0.05)
		Negative Control < pH 4 (p < 0.05)
		pH 5.6 < pH 4 (p < 0.01)
		Negative Control < pH 7 (p < 0.05)
		pH 5.6 < pH 7 (p < 0.05)

*Statistical analyses were performed using Kruskal Wallis tests with Dunn's Multiple Comparisons Post Tests. All treatment groups were compared in a Kruskal Wallis test, and if that indicated significant differences were present between groups, each treatment group was compared to every other treatment group in post testing. Only statistically significant post test results are shown. Any treatment group comparisons not listed were not significantly different from one another (p > 0.05).

### Immunohistochemistry

**Figure 7:**
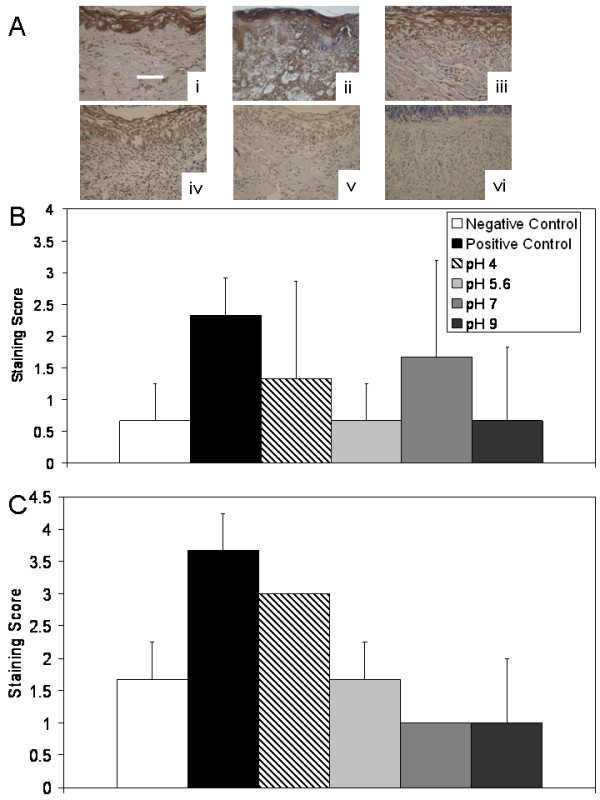
**Immunohistochemical detection of TNF-α in biopsies of DNCB-induced rashes treated with various nanocrystalline silver-derived solutions**. (A) Representative images are shown for immunohistochemical detection of TNF-α after 72 h treatment of negative controls with distilled water (i), and DNCB-induced porcine contact dermatitis rashes with distilled water (positive controls) (ii), or nanocrystalline silver-derived solutions generated at starting pHs of 4 (iii), 5.6 (iv), 7 (v), or 9 (vi). The scale bar in A represents 100 μm. Staining for TNF-α appears brown, while the cell nuclei are counterstained purple using haematoxylin. Immunohistochemical staining scores for TNF-α are shown after 24 h (B) and 72 h (C) of treatment as described above. Statistical analyses, which were performed using one-way ANOVAs with Tukey-Kramer Multiple Comparisons post tests, are shown in Table 4. Error bars represent standard deviations.

Figure 7 shows an example of the immunohistochemical images obtained in Panel (A): Representative images of immunohistochemical staining for TNF-α after 72 hours of treatment are shown. Immunohistochemical staining scores are shown after 24 h and 72 hours of treatment in Panels (B) and (C), respectively. Table 4 shows statistical analysis of the staining scores for all cytokines and growth factors analyzed. Negative controls showed some staining in the epidermis throughout the experiment, but otherwise had low TNF-α levels. Positive controls showed widespread TNF-α staining, which increased in intensity during the treatment period. Of the treatment groups, animals treated with pH 7 solutions showed the strongest staining for TNF-α at 24 hours, however this trend did not reach significance. At 72 hours, staining for TNF-α was somewhat increased with pH 4 and pH 5.6 treatments, particularly in the newly forming epidermis, but not to the levels observed in positive controls. In particular, pH 5.6 treated animals still had significantly lower scores than positive controls (p < 0.05). TNF-α staining appeared to decrease with increasing pH of treatment at 72 hours, with animals treated with pH 7 and 9 solutions having significantly lower staining scores for TNF-α relative to positive controls (p < 0.01) and pH 4 treated animals (p < 0.05) (see Table 4).

**Table 4:** Statistical analysis of immunohistochemical staining scores*.

Cytokine/Growth Factor	Time (h)	ANOVA	Post Test Results
TNF-α	24 h	p = 0.3582	No significant differences.
TNF-α	72 h	p = 0.0004	pH 7 < pH 4 (p < 0.05)
			pH 9 < pH 4 (p < 0.05)
			pH 7 < Positive Control (p < 0.01)
			pH 9 < Positive Control (p < 0.01)
			Negative Control < Positive Control (p < 0.05)
IL-8	24 h	p = 0.1742	No significant differences.
IL-8	72 h	p = 0.0492	pH 5.6 < Positive Control (p < 0.05)
IL-4	24 h	p = 0.0248	Negative Control < pH 9 (p < 0.05)
IL-4	72 h	p = 0.0026	Negative Control < pH 4 (p < 0.05)
			Negative Control < pH 5.6 (p < 0.01)
			Negative Control < pH 7 (p < 0.01)
			Negative Control < pH 9 (p < 0.01)
EGF	24 h	p = 0.0031	Negative Control < pH 9 (p < 0.01)
			Positive Control < pH 9 (p < 0.05)
			pH 4 < pH 9 (p < 0.01)
			pH 7 < pH 9 (p < 0.01)
EGF	72 h	p = 0.0199	No significant differences.
KGF	24 h	p = 0.0039	Negative Control < pH 9 (p < 0.05)
			Positive Control < pH 9 (p < 0.05)
			pH 4 < pH 9 (p < 0.05)
			pH 7 < pH 9 (p < 0.05)
			pH 7 < pH 5.6 (p < 0.05)
KGF	72 h	p = 0.1310	No significant differences.
KGF-2	24 h	p = 0.0105	pH 7 < pH 9 (p < 0.05)
			Negative Control < pH 9 (p < 0.05)
KGF-2	72 h	p = 0.0173	Negative Control < pH 5.6 (p < 0.05)
			Negative Control < pH 7 (p < 0.05)
IL-10	24 h	p = 0.6472	No significant differences.
IL-10	72 h	p = 0.8397	No significant differences.

*Statistical analyses were performed using one-way ANOVAs with Tukey-Kramer Multiple Comparisons Post Tests. All treatment groups were compared in an ANOVA, and if the ANOVA indicated significant differences were present between groups, each treatment group was compared to every other treatment group in post testing. Only statistically significant post test results are shown. Any treatment group comparisons not listed were not significantly different from one another (p > 0.05).

**Figure 8:**
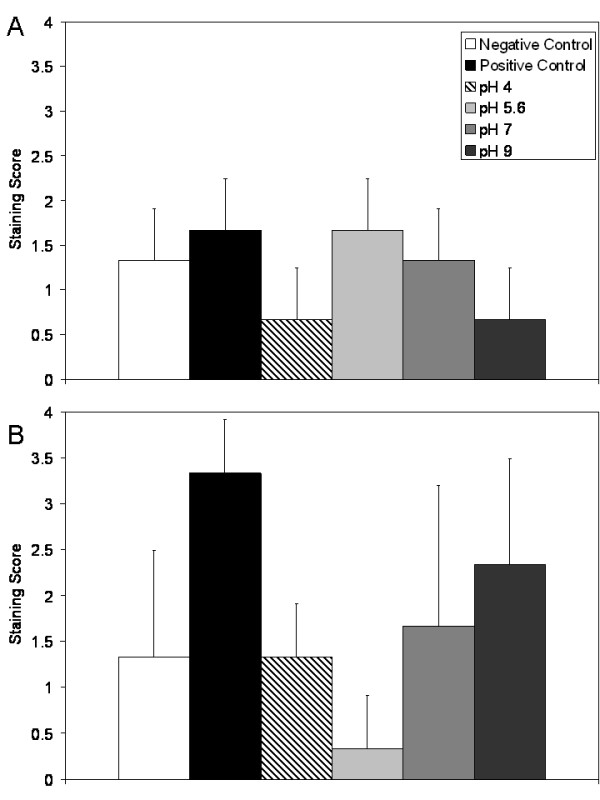
**Immunohistochemical detection of IL-8 in biopsies of DNCB-induced rashes treated with various nanocrystalline silver-derived solutions**. Immunohistochemical staining scores for IL-8 are shown after 24 h (A) and 72 h (B) of treatment of negative controls with distilled water, and DNCB-induced porcine contact dermatitis rashes with distilled water (positive controls), or nanocrystalline silver-derived solutions generated at starting pHs of 4, 5.6, 7, or 9. Statistical analyses, which were performed using one-way ANOVAs with Tukey-Kramer Multiple Comparisons post tests, are shown in Table 4. Error bars represent standard deviations.

Figure 8 shows immunohistochemical staining scores for IL-8 after 24 and 72 hours of treatment in Panels (A) and (B), respectively. As with TNF-α, negative controls showed some staining for IL-8 in the epidermis, but low levels in the dermis throughout the experiment. Positive controls, and pH 5.6 and 7 solution-treated animals, showed mild increases in IL-8 staining relative to negative controls at 24 hours, while pH 4 and 9 treated animals showed lower levels of staining. However, this trend did not reach significance (see Table 4). At 72 hours, positive controls showed strong staining for IL-8 throughout the epidermis and in a cell-associated fashion in the dermis. Animals treated with pH 4, 5.6, and 7 solutions showed low staining for IL-8 at this time point, with the pH 5.6 solution treated animals having significantly lower staining scores relative to the positive controls (p < 0.05). Interestingly, animals treated with pH 9 solutions showed stronger staining for IL-8 in the epidermis at 72 hours, although this was not as dark as the staining present in the positive controls.

**Figure 9:**
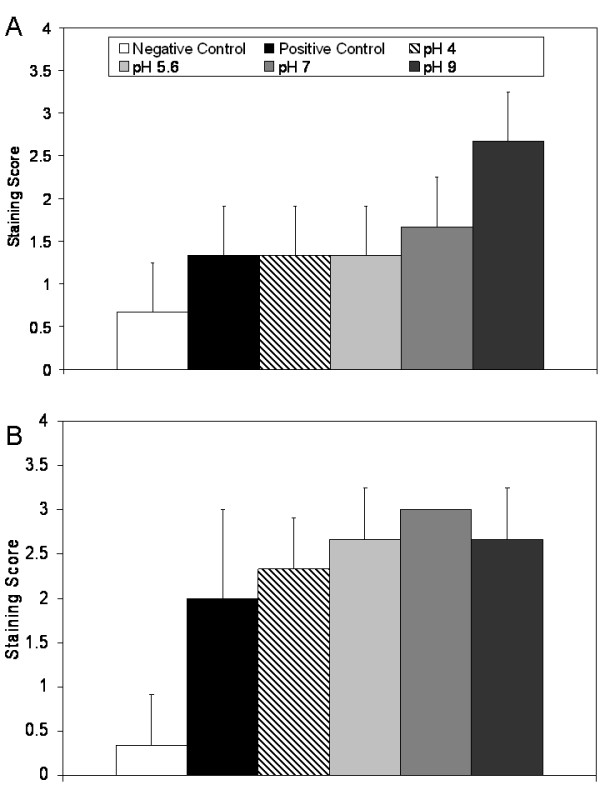
**Immunohistochemical detection of IL-4 in biopsies of DNCB-induced rashes treated with various nanocrystalline silver-derived solutions**. Immunohistochemical staining scores for IL-4 are shown after 24 h (A) and 72 h (B) of treatment of negative controls with distilled water, and DNCB-induced porcine contact dermatitis rashes with distilled water (positive controls), or nanocrystalline silver-derived solutions generated at starting pHs of 4, 5.6, 7, or 9. Statistical analyses, which were performed using one-way ANOVAs with Tukey-Kramer Multiple Comparisons post tests, are shown in Table 4. Error bars represent standard deviations.

Figure 9 shows immunohistochemical staining scores for IL-4 after 24 and 72 hours of treatment in Panels (A) and (B), respectively. Negative controls showed low levels of staining for IL-4 throughout the study, with only mild cell-specific staining in the dermis. Positive controls and animals treated with pH 4, 5.6, and 7 solutions showed low levels of widespread staining at 24 hours of treatment. However, animals treated with pH 9 solutions showed stronger staining at 24 hours of treatment. This was the only treatment group to have significantly stronger staining than the negative controls at 24 hours (p < 0.05, see Table 4). At 72 hours of treatment, mild increases in IL-4 staining were observed in some keratinocytes of the positive controls and pH 4 treated solutions, with the pH 4 treated solutions having significantly stronger staining than the negative controls (p < 0.05). Strong staining was observed in the keratinocytes throughout the newly re-epithelialised tissues which had been treated with pH 5.6, 7, and 9 solutions. In these tissues, cell-associated staining was also observed in the dermis, most likely in fibroblasts. These tissues all had significantly stronger staining than negative controls (p < 0.01).

**Figure 10:**
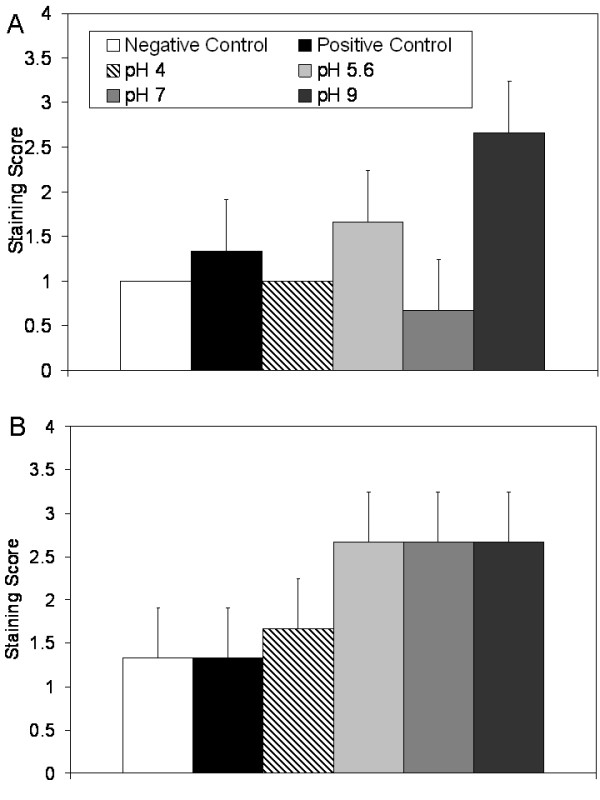
**Immunohistochemical detection of EGF in biopsies of DNCB-induced rashes treated with various nanocrystalline silver-derived solutions**. Immunohistochemical staining scores for EGF are shown after 24 h (A) and 72 h (B) of treatment of negative controls with distilled water, and DNCB-induced porcine contact dermatitis rashes with distilled water (positive controls), or nanocrystalline silver-derived solutions generated at starting pHs of 4, 5.6, 7, or 9. Statistical analyses, which were performed using one-way ANOVAs with Tukey-Kramer Multiple Comparisons post tests, are shown in Table 4. Error bars represent standard deviations.

Figure 10 shows immunohistochemical staining scores for EGF after 24 and 72 hours of treatment in Panels (A) and (B), respectively. Negative controls showed low staining for EGF throughout the experiment, with some cell specific staining present in the dermis. Positive controls showed mild widespread staining for EGF at 24 h, while pH 4 and pH 7 solution treatments resulted in low levels of staining, which was present in areas of tissue damage. Animals treated with pH 5.6 solutions showed somewhat stronger staining, both in the damaged epidermis and in the dermis where re-epithelialisation would later take place. Animals treated with pH 9 solutions showed staining in the damaged epidermis, but much stronger cell associated staining in the newly forming epidermis. This staining was significantly stronger than all other groups except the pH 5.6 solution treated animals (see Table 4). At 72 hours, all treatment groups, but especially those animals treated with pH 5.6 or higher, showed strong EGF staining in keratinocytes of the newly formed epidermis, as well as some staining in the dermis associated with cells that are most likely fibroblasts. Positive controls continued to show only low levels of widespread staining. While ANOVA testing indicated that there were significant differences between groups, the post tests did not identify differences between individual groups (see Table 4).

**Figure 11:**
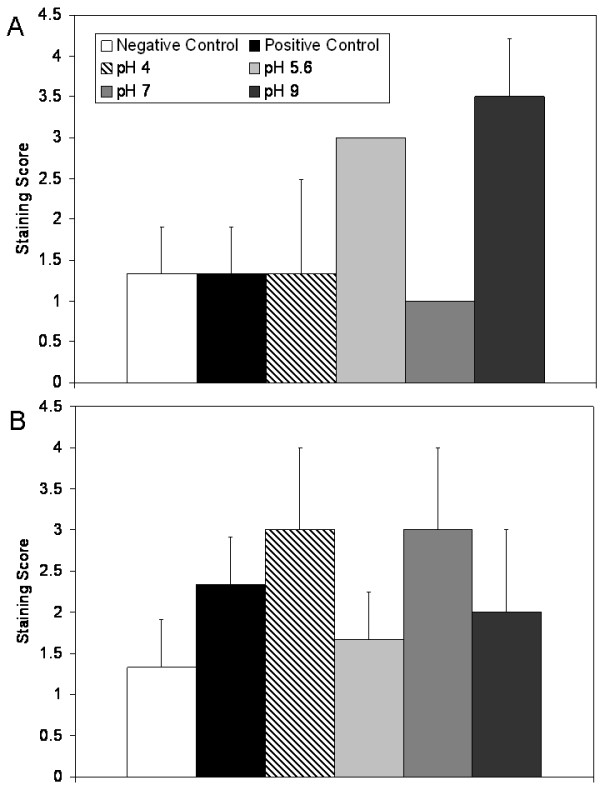
**Immunohistochemical detection of KGF in biopsies of DNCB-induced rashes treated with various nanocrystalline silver-derived solutions**. Immunohistochemical staining scores for KGF are shown after 24 h (A) and 72 h (B) of treatment of negative controls with distilled water, and DNCB-induced porcine contact dermatitis rashes with distilled water (positive controls), or nanocrystalline silver-derived solutions generated at starting pHs of 4, 5.6, 7, or 9. Statistical analyses, which were performed using one-way ANOVAs with Tukey-Kramer Multiple Comparisons post tests, are shown in Table 4. Error bars represent standard deviations.

Figure 11 shows immunohistochemical staining scores for KGF after 24 and 72 hours of treatment in Panels (A) and (B), respectively. Negative controls showed similar levels of staining for KGF in the epidermis throughout the study. Positive controls showed low staining for KGF at 24 hours, as did pH 4 and 7 solution-treated animals. Animals treated with pH 5.6 and 9 solutions showed strongly increased staining, particularly in the epidermis. Animals treated with pH 5.6 solutions had significantly stronger staining than animals treated with pH 7 solutions (p < 0.05), while animals treated with pH 9 solutions had significantly stronger staining than all other groups except animals treated with pH 5.6 solutions (p < 0.05, see Table 4). By 72 hours, differences in tissue staining appeared to level out, with medium-strength cell-specific staining occurring in the epidermis of the positive controls and all treatment groups. No significant differences were found at this time point (see Table 4).

**Figure 12:**
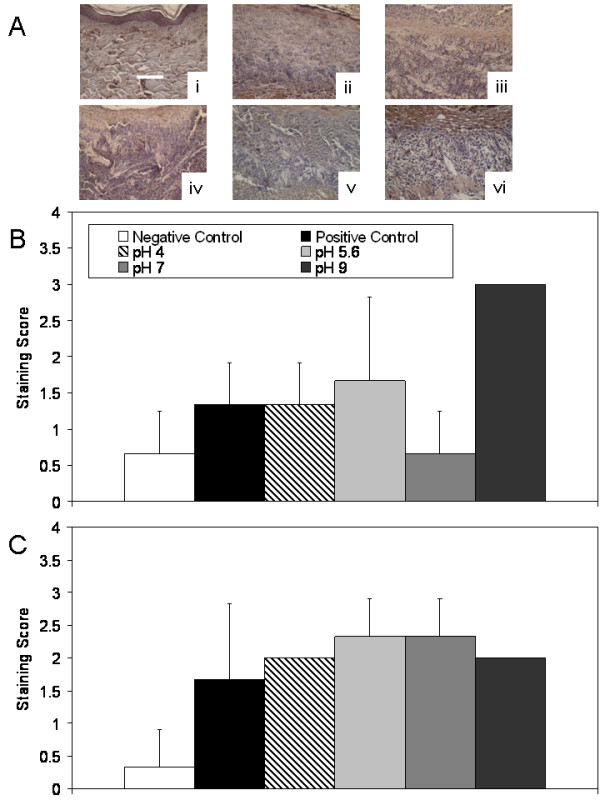
**Immunohistochemical detection of KGF-2 in biopsies of DNCB-induced rashes treated with various nanocrystalline silver-derived solutions**. (A) Representative images are shown for immunohistochemical detection of KGF-2 after 24 h treatment of negative controls with distilled water (i), and DNCB-induced porcine contact dermatitis rashes with distilled water (positive controls) (ii), or nanocrystalline silver-derived solutions generated at starting pHs of 4 (iii), 5.6 (iv), 7 (v), or 9 (vi). The scale bar in A represents 100 μm. Staining for KGF-2 appears brown, while the cell nuclei are counterstained purple using haematoxylin. Immunohistochemical staining scores for KGF-2 are shown after 24 h (B) and 72 h (C) of treatment as described above. Statistical analyses, which were performed using one-way ANOVAs with Tukey-Kramer Multiple Comparisons post tests, are shown in Table 4. Error bars represent standard deviations.

Figure 12 shows another example of the immunohistochemical images obtained: Representative images of immunohistochemical staining for KGF-2 after 24 hours of treatment are shown in Panel (A). Immunohistochemical staining scores for KGF-2 after 24 and 72 hours of treatment are shown in Panels (B) and (C), respectively. Negative controls showed some cell-specific staining for KGF-2 in the epidermis and dermis at 24 hours, but minimal staining at 72 hours. Positive controls showed low levels of widespread staining throughout the study. At 24 hours, animals treated with pH 4 and 5.6 solutions showed low levels of staining for KGF-2 near tissue surfaces, while animals treated with pH 7 solutions showed minimal staining for KGF-2. Animals treated with pH 9 solutions showed strong cell-associated staining for KGF-2, particularly in the keratinocytes of the newly forming epidermis, but also to a lesser extent in the dermis, perhaps in fibroblasts. This staining was significantly higher than that in negative controls or pH 7 solution treated animals (p < 0.05). At 72 hours, all animals receiving silver-containing treatments showed this type of strong cell-associated staining. In animals treated with pH 5.6 or 7 solutions, the staining was significantly stronger than that seen in the negative controls (p < 0.05, see Table 4).

**Figure 13:**
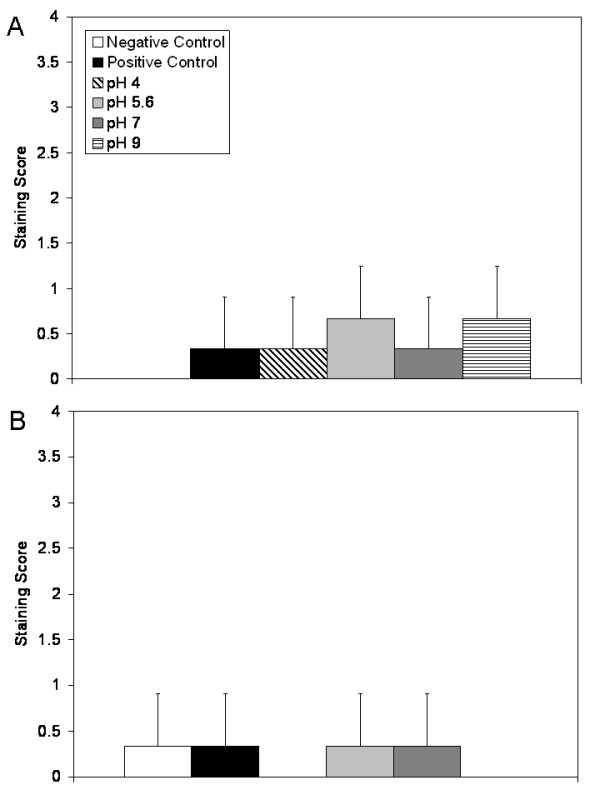
**Immunohistochemical detection of IL-10 in biopsies of DNCB-induced rashes treated with various nanocrystalline silver-derived solutions**. Immunohistochemical staining scores for IL-10 are shown after 24 h (A) and 72 h (B) of treatment of negative controls with distilled water, and DNCB-induced porcine contact dermatitis rashes with distilled water (positive controls), or nanocrystalline silver-derived solutions generated at starting pHs of 4, 5.6, 7, or 9. Statistical analyses, which were performed using one-way ANOVAs with Tukey-Kramer Multiple Comparisons post tests, are shown in Table 4. Error bars represent standard deviations.

Immunohistochemical analysis of IL-10 did not show strong staining in any of the groups tested at any time point (see Figure 13), and there were no significant differences between groups (see Table 4), although staining was present around blood vessels.

### Average Silver and Calcium Delivered

The average daily total of silver delivered via the nanocrystalline silver-derived solutions to each treatment group was as follows: 1.88 ± 0.59 mg (pH 4), 0.28 ± 0.11 mg (pH 5.6), 0.20 ± 0.08 mg (pH 7), 0.27 ± 0.15 mg (pH 9). There were significant differences between treatment groups (p = 0.0011), with animals in the pH 4 solution treatment group receiving significantly higher (p < 0.01) total silver per day than all other groups. There were no significant differences in silver delivered to the remaining groups. Based on the quantities of Ca(OH)_2 _used to generate solutions, and the volumes delivered per day to the animals, animals in the pH 7 and pH 9 solution groups received approximately 0.004 mg and 0.019 mg Ca^+ ^per day, respectively.

## Discussion

In this study, contact dermatitis was induced using DNCB in a porcine model. This is a well-established model of inflammation, since DNCB-induced dermatitis is the prototype of T-cell mediated delayed-type hypersensitivity reactions[1,2,21]. It is also a clinically relevant model, due to the similarities between porcine and human skin[22-24]. Consistent strong inflammation was observed at Day 0 in this study. Negative controls did not appear to be impacted by treatment with distilled water, while positive controls did not show signs of improvement over 72 hours.

This study shows that solutions derived from nanocrystalline silver have some or all of the anti-inflammatory properties of nanocrystalline silver dressings previously reported[2,3,6,7], depending on dissolution conditions. Nanocrystalline silver-derived solutions were able to reduce visual and histological signs of inflammation. This occurred in conjunction with induction of apoptosis in infiltrating inflammatory cells. Apoptosis of these cells may have led to the observed reduction in expression of TNF-α and IL-8, which are both key mediators of the inflammatory response[25,26]. Apoptosis is involved with eliminating inflammatory cells from inflamed tissues[25], and compounds which induce apoptosis, including noble metals[11-14,27-29], are beneficial in the treatment of inflammatory diseases. Thus, induction of apoptosis in inflammatory cells by nanocrystalline silver-derived solutions appears to be a key factor for their anti-inflammatory activity. Apoptosis induction may have, in part, been regulated by the observed increased expression of IL-4, an anti-inflammatory cytokine which induces apoptosis of neutrophils and macrophages, and downregulates the effects of IL-1, TNF-α, IL-6, and IL-8 on macrophages[30-33]. IL-10 did not appear to be involved in the anti-inflammatory effect seen. This was also observed previously in a study using nanocrystalline silver dressings[7], but differs from a study using silver nanoparticles to treat murine thermal injuries[34]. As with nanocrystalline silver dressings[7], upregulation of EGF, KGF, and KGF-2 was observed with nanocrystalline silver-derived treatments. EGF promotes keratinocyte migration, enhancing re-epithelialisation[35-37]; enhances formation of granulation tissue; and stimulates fibroblast motility[38]. KGF and KGF-2 both stimulate proliferation and migration of keratinocytes; promote detoxification of reactive oxygen species (ROS), protecting keratinocytes from ROS-induced apoptosis[37,39]; and are involved indirectly with granulation tissue formation[39,40]. Thus, upregulation of these growth factors may partially explain the enhanced re-epithelialisation rates and pro-healing activity observed with nanocrystalline silver-derived solutions.

Solutions generated at a starting pH of 4 showed only mild visual improvements, with the only significant reduction in oedema relative to positive controls occurring at 48 h. However, histology showed that re-epithelialisation began to occur within two days of treatment, and that there was reduced inflammatory cell infiltration after this time point. There were no significant trends related to MMP expression or activity. At 24 hours, apoptosis did not appear to be induced at tissue surfaces. However, deeper in the dermis (which is normally near-acellular but which had been infiltrated by inflammatory cells) large numbers of apoptotic cells were observed, with visible blebbing. Since apoptosis levels were no higher than those observed in positive controls in the epidermis, this suggests that very little Ag^+ ^was present in the solutions delivered to the animals, as previous studies have suggested that Ag^+ ^produces indiscriminate apoptosis induction near tissue surfaces[6]. This suggests that, since there was high total silver in solution, most of the silver being delivered was inactive. At pH 4, high quantities of Ag^+ ^may be dissolved into solution initially, which then react with the carbonic acid present to form silver carbonates. This appears to be confirmed by the occasional presence of visible white flakes in solution. Thus, the Ag^+ ^and any higher oxidation state silver released may be inactivated, creating a gradient for the release of additional silver species, including the anti-inflammatory silver species, which are then able to produce the anti-inflammatory/pro-healing effects observed, including the selective apoptosis of inflammatory cells. Previous studies have demonstrated apoptosis induction selective to inflammatory cells of the dermis[2,3,6]. Also, a recent study suggested that this action was via silver interactions with cells close to tissue surfaces which then release signals resulting in an anti-inflammatory series of events including apoptosis induction in inflammatory cells[7]. Treatment with pH 4 solutions resulted in somewhat decreased TNF-α expression relative to positive controls at 72 hours, but this was not as considerable as other treatment groups. However, IL-8 expression was lower than that of positive controls at 72 hours, showing that treatment with pH 4 solutions may be capable of reducing pro-inflammatory cytokine expression. pH 4 treatments did not increase IL-4 expression as strongly as other treatment groups, which may in part explain the weaker anti-inflammatory effect observed. This also suggests that other signalling molecules, not yet identified, were responsible for the significant apoptosis induction observed in the deep dermis. However, pH 4 treatments did result in mildly increased EGF expression by 72 hours, a mild increase in KGF expression at 24 hours, and increased KGF-2 expression at 24 hours in areas where re-epithelialisation would later occur beneath the scab, followed by a mild increase in KGF-2 expression at 72 hours in the newly forming epidermis. The enhanced expression of these growth factors explains, at least in part, the pro-healing activity observed, such as enhanced rate of re-epithelialisation and protection of keratinocytes from apoptosis induction.

Animals treated with solutions generated at a starting pH of 5.6 showed similar mild visual improvements to pH 4 treatments. They had no significant decreases in erythema or oedema. Histologically, they were the slowest to begin re-epithelialisation and to show signs of decreased leukocyte infiltration, both of which occurred between 48 and 72 hours of treatment. However, this was still enhanced relative to positive controls. pMMP-2 expression was significantly decreased relative to positive controls at 24 hours, and similar trends (not significant) were observed with pMMP-9, aMMP-9, and aMMP-2. Interestingly, apoptosis induction was not observed at 24 hours of treatment, meaning that the reduction of aMMP-2 levels could not have occurred solely through apoptosis of inflammatory cells, and therefore may have occurred via modulation of cellular output and activation of these molecules. Apoptosis induction was examined at 24 hours, since previous studies have shown apoptosis induction at this time point [6,7] using nanocrystalline silver dressings. However, it seems likely that with pH 5.6 treatments, apoptosis induction in inflammatory cells occurred later, and could perhaps have been detected at 48 hours, since reduced inflammatory cell presence and re-epithelialisation were not observed until 72 hours. In addition, a study of contaminated porcine wounds demonstrated increased apoptosis in inflammatory cells at 48 hours with nanocrystalline silver dressings[3]. The pH 5.6 solution-treated animals showed poor modulation of pro-inflammatory cytokine expression relative to the other treatment groups, with TNF-α expression increased at 72 hours, and IL-8 expression increased at 24 hours. However, the expression of TNF-α and IL-8 in this group was still lower than that of positive controls, with IL-8 expression at 72 hours being significantly lower than positive controls. The expression of anti-inflammatory cytokine IL-4 was increased at 72 hours, which again suggests that apoptosis induction may have occurred between 24 and 72 hours in inflammatory cells. With pH 5.6 treatments, modulation of growth factors related to re-epithelialisation was observed: EGF expression was increased at 72 hours; KGF expression was increased at 24 hours, but decreased somewhat at 72 hours; and KGF-2 was expressed at 24 hours in areas where re-epithelialisation would later occur, and was expressed at 72 hours in newly formed epidermis. Since growth factor expression was not delayed, the delayed re-epithelialisation appears to have been related to the late induction of apoptosis in inflammatory cells of these tissues. The delayed healing in this group relative to other treatment groups may be due to Ag^+ ^levels in solution. In distilled water, Ag^+ ^would be less able to react to form inactive/insoluble species than in treatments containing carbonates or hydroxyls, and that, in combination with the low total silver dissolved at pH 5.6, might result in a poor gradient for drawing active silver species, including anti-inflammatory species, into solution, while the Ag^+ ^present could counteract the pro-healing/anti-inflammatory activity to some degree, delaying healing. The early upregulation of KGF may have protected keratinocytes from Ag^+^-induced apoptosis. Interestingly, solutions generated in distilled water have the highest antimicrobial activity, corroborating the idea that most of the silver present in solution is positively charged (unpublished data).

Solutions generated at a starting pH of 7 showed visual improvements during the treatment period, with significant reductions in oedema at 48 hours. Histopathology showed that re-epithelialisation had begun by 48 hours, with greatly decreased inflammatory cell infiltration occurring by 72 hours. However, the epidermis was very thick with deep ridges. While some researchers have suggested that this tissue morphology is beneficial[41], as it indicates that the newly forming tissues are well anchored and less likely to dehisce, others have suggested that this tissue morphology is caused by chronic mechanical shear[42], which, in this case, would be due to animals attempting to scratch their backs or remove their dressings, possibly due to discomfort caused by the treatment. The latter seems unlikely, since animals treated with pH 9 solutions did not demonstrate epidermal thickening or extended rete ridges, although they were exposed to the same quantity of silver and even more calcium hydroxide. If the treatment was irritating, the same effect should have been observed in the pH 9 treatment group. A third possibility is that the epidermal tissue only appeared to be thicker in this group due to angled slices through the tissue during slide preparation. While a fourth possibility is that hyperkeratinisation was occurring, this seems unlikely, considering the low expression of KGF and KGF-2 at 24 hours relative to other treatment groups. There was a trend towards decreased MMP expression and activation with pH 7 treatments, but this was not significant. At 72 hours, only one of three animals showed strong MMP expression, while positive controls had two out of three animals showing strong MMP expression. Although strong apoptosis induction was observed in the dermis with pH 7 treatments, some apoptosis induction was also observed in the epidermis, suggesting either a less selective or less protective effect with this treatment. One possible explanation for this is that there may not have been enough hydroxyls present in the solution to bind to all the Ag^+ ^released, and thus the Ag^+ ^caused apoptosis in the epidermis. However, if this was the case, a similar observation would have been expected with the pH 5.6 treatment, where high levels of Ag^+ ^seem more likely. Furthermore, calculations based on the amount of silver and amount of calcium hydroxide present in the solutions suggest that there should have been sufficient hydroxyl ions to react with the Ag^+^. It also should be noted that the apoptosis induction in the epidermis was not as strong as that observed previously with silver nitrate treatments (which contain Ag^+ ^only)[6], and that the increased expression observed did not reach statistical significance. Expression of pro-inflammatory molecules was modulated via pH 7 treatments, with TNF-α downregulated by 72 hours, and IL-8 expression low at both 24 and 72 hours. In addition, the expression of anti-inflammatory cytokine IL-4 was increased at 72 hours, along with EGF. Thus pH 7 solutions provide active silver species which generate anti-inflammatory/pro-healing activity. However, expression of KGF and KGF-2 was low at 24 hours compared to other treatment groups. Since these molecules are involved with protection of keratinocytes from apoptosis, this may partially explain why some apoptosis occurred in the epidermis with this treatment.

Solutions generated at a starting pH of 9 showed the most visual improvement, with significant reductions in both erythema and oedema scores within two days of treatment. Histologically, re-epithelialisation occurred the earliest, and the most normal tissue morphology was observed at 72 hours. Apoptosis induction was observed in the deep dermis at 24 hours, but not in the newly forming epidermis. The induction levels were not as high as those seen with pH 4 or pH 7 treatments, but since re-epithelialisation was already occurring in some of the pH 9 treated animals, it is possible that the peak of apoptosis induction had already passed at 24 hours. TNF-α expression was low throughout the treatment period, but, interestingly, IL-8 expression increased over time, although not to the levels observed with the positive controls. Expression of IL-4 was increased throughout the treatment period, at least partially explaining the anti-inflammatory activity observed. In addition, EGF expression was increased throughout the treatment period, while KGF expression was increased at 24 h, and KGF-2 expression was increased throughout the experiment, particularly in newly forming epidermis and fibroblasts. This early increased expression of growth factors involved in re-epithelialisation and formation of granulation tissue explains, at least in part, the enhanced pro-healing activity observed with the pH 9 treatments. Overall, these results suggest that at higher pHs more of the total silver in solution is active anti-inflammatory species, perhaps Ag^(0) ^clusters[9]. The Ag^+ ^released into solution along with these species may react with hydroxyl ions from the calcium hydroxide in solution[43] causing their re-precipitation, preventing them from inhibiting healing, and resulting in a gradient towards the release of more anti-inflammatory silver. The levels of Ag^+ ^in nanocrystalline silver containing solutions generated at different pHs will be tested in the future.

It should be noted that serum and wound fluids contain 160-185 mg/L[44] calcium. The daily calcium provided in the pH 7 and 9 treatments was thus <0.0025% and <0.012% of the calcium load per litre of blood, respectively. Therefore, it is unlikely that the calcium provided was a significant contributor to the effects observed.

## Conclusions

Overall, nanocrystalline silver-derived solutions appear to have anti-inflammatory/pro-healing activity, particularly when generated at a starting pH of 9. Since this activity does not correlate with total silver, solutions generated under different conditions may have varying concentrations of different silver species, only some of which have anti-inflammatory activity. Future studies will focus on improving understanding of which species have anti-inflammatory activity and what conditions are necessary to generate or dissolve these species. Nanocrystalline silver-derived solutions show promise for a variety of anti-inflammatory treatment applications.

## Competing interests

The authors declare that they have no competing interests.

## Authors' contributions

PN participated in the conception, design, and coordination of the study, including obtaining ethics approval for the study, and all materials needed for the study. PN created the DNCB-induced rashes on the animals, and participated in sample collection and preservation, and treatment of the animals. PN performed the histological analysis, zymography, apoptosis detection, immunohistochemistry, statistics, and drafted the paper. RB participated in the conception and design of the study, and provided critical revision of the manuscript. JW provided assistance with zymography and apoptosis detection, and provided critical revision of the manuscript. ET provided equipment and lab space for histological analysis, zymography, apoptosis detection, and immunohistochemistry, and provided critical revision of the manuscript. All authors read and approved the final manuscript.
